# Pin in Bronchus—Report of a Case

**Published:** 1911-12

**Authors:** F. G. Hodgson

**Affiliations:** Atlanta, Ga.


### PIN IN BRONCHUS—REPORT OF A CASE.

By F. G. Hodgson, M. D., Atlanta, Ga.

April 9th, 1910. Baby girl 10 months old was taken to see my colleague, Dr. John S. Hurt. Mother said the baby had been playing with a doll which had a large brass-headed pin in its dress. The baby was found to be choking and coughing and blood-stained saliva or mucous came out of its mouth. On examining the doll the pin was found to be missing. The mother saw one doctor, who made light of the story and said the baby was all right. Not satisfied, she saw Dr. Hurt, who took an X-ray picture and located the pin lying in the right bronchus, head downward.

Operation: Ether anaesthesia; incision in mid-line of neck just above suprasternal notch, 1 inch in length; deepened until trachea was exposed; trachea opened; very narrow dressing, forceps inserted with some difficulty on account of the narrow lumen of the trachea in a child so young; pin located and removed; trachea allowed to fall together without suture; subcutaneous suture No. 1 plain cat-gut; few strands of silkworm gut used as drain in lower angle. Recovery uneventful. Highest temperature 100. Slight tracheal cough. Voice a little husgy for a few days.

